# Global Transcriptomic Analysis Reveals Insights into the Response of ‘Etrog’ Citron (*Citrus medica* L.) to *Citrus Exocortis Viroid* Infection

**DOI:** 10.3390/v11050453

**Published:** 2019-05-17

**Authors:** Yafei Wang, Jiaxing Wu, Yuanjian Qiu, Sagheer Atta, Changyong Zhou, Mengji Cao

**Affiliations:** 1 National Citrus Engineering Research Center, Citrus Research Institute, Southwest University, Chongqing 400712, China; yafeiwang2019@163.com (Y.W.); wjx0117@email.swu.edu.cn (J.W.); qyjian2015@163.com (Y.Q.); 2 Academy of Agricultural Sciences, Southwest University, Chongqing 400715, China; 3 Department of Plant Protection, Faculty of Agricultural Sciences, Ghazi University, Dera Ghazi Khan 32200, Pakistan; satta@gudgk.edu.pk

**Keywords:** *Citrus exocortis viroid* (CEVd), ‘Etrog’ citron, differentially expressed genes, transcriptome, RNA silencing, innate immunity

## Abstract

*Citrus exocortis viroid* (CEVd) is the causal agent of citrus exocortis disease. We employed CEVd-infected ‘Etrog’ citron as a system to study the feedback regulation mechanism using transcriptome analysis in this study. Three months after CEVd infection, the transcriptome of fresh leaves was analyzed, and 1530 differentially expressed genes were detected. The replication of CEVd in citron induced upregulation of genes encoding key proteins that were involved in the RNA silencing pathway such as Dicer-like 2, RNA-dependent RNA polymerase 1, argonaute 2, argonaute 7, and silencing defective 3, as well as those genes encoding proteins that are related to basic defense responses. Many genes involved in secondary metabolite biosynthesis and chitinase activity were upregulated, whereas other genes related to cell wall and phytohormone signal transduction were downregulated. Moreover, genes encoding disease resistance proteins, pathogenicity-related proteins, and heat shock cognate 70 kDa proteins were also upregulated in response to CEVd infection. These results suggest that basic defense and RNA silencing mechanisms are activated by CEVd infection, and this information improves our understanding of the pathogenesis of viroids in woody plants.

## 1. Introduction

Viroids are small, circular, infectious RNAs that do not encode any protein, and their genomes range from 246 to 433 nucleotides [1,2]. Viroids can replicate autonomously in higher plants, and they can infect important economically significant crops and cause severe diseases [3]. They are the causative agents of various diseases affecting herbaceous and woody plants as well as agronomic and ornamental plants around the world and can cause chlorosis, leaf deformation, stunting, and even plant death on sensitive hosts [4]. RNA silencing is an important defense mechanism for plants to cope with RNA virus and viroid infections. Viroids can function as precursors for small interfering RNAs (siRNAs), and viroid-derived siRNAs involved in post-transcriptional gene silencing (PTGS) cause RNA silencing of the host mRNA to induce disease symptoms in higher plants [5]. Studies have been conducted to detect siRNAs in PSTVd-infected tobacco and tomato plants and demonstrate that viroids are activators and targets for RNA silencing [6,7]. The plant’s Dicer-like proteins (DCLs) fragment double-stranded RNAs into small RNAs that mediate argonaute proteins (AGOs) to inhibit RNA viruses and viroids [5]. In addition, pathogen-associated molecular patterns (PAMPs)-triggered immunity (PTI) and effector-triggered immunity (ETI) are plant innate immune mechanisms. However, because there are no clear molecular patterns, the contribution of PTI and ETI in plant defenses against RNA viruses and viroids remains unclear [8].

As demonstrated by various types of plant pathogens [9,10], an overall analysis of gene expression patterns in plants infected with viroids is important for understanding pathogenesis and developing disease management strategies. Previous studies have used differential display [11] and microarray technology [12,13] to obtain alterations of host gene expression following viroid infections. Viroid infections affect biological functions such as stress and defense response, chloroplast biogenesis, cell wall structure, and protein metabolism [12,13,14]. Genes involved in various plant hormone biosyntheses and signal transduction also exhibit altered expression during viroid infections [12,15]. Most of the host genes changed by viroid infections are also sensitive to other different pathogens, suggesting that some common regulatory networks are associated with the induction of viroid diseases. There is currently insufficient data to construct a clear network to explain the progression of viroid diseases. Recently, RNA-sequencing (RNA-seq) has served as a novel platform for global analysis of transcriptomes, which is superior to microarray technology in detecting range sensitivity and can significantly reduce costs [16]. At the same time, RNA-seq has higher reproducibility and lower sample requirements [17,18], making it a powerful tool for genome-wide expression studies.

Transcriptome studies describing plant-viroid interactions are limited, including tomato and potato that are infected with *potato spindle tuber viroid* (PSTVd) [12,19], peaches infected with *peach latent mosaic viroid* (PLMVd) [20], hops and cucumbers infected with *hop stunt viroid* (HSVd) [21,22]. These studies have indicated that the expression levels of genes related to plant immune responses, plant hormone signal transduction, secondary metabolism, protein metabolism, and cell wall are altered after viroid infection, thereby providing new insights into how hosts respond to viroid infections. Recently, a comprehensive analysis of hops infected with *hop latent viroid* (HLVd) and *citrus bark cracking viroid* (CBCVd) has been performed [23]. Numerous hop transcripts were found to have nucleotide sequence similarity to viroid-derived small RNAs involved in RNA interference, and some pathogenesis-related genes were also highly expressed in viroid-infected hops [23]. These researches are mostly concentrated on herbaceous hosts, but the natural infection reports of viroids are more common in woody plants such as citrus. Therefore, comparative analysis of some other viroid-host combinations, especially the interaction between viroids and their woody hosts, may be helpful in further understanding the pathogenesis of viroids.

*Citrus exocortis viroid* (CEVd) is the causal agent of citrus exocortis and affects trifoliate orange [*Poncirus trifoliata* (L.) Raf.] and its hybrids, which are all widely used as rootstocks in commercial orchards [24,25,26,27]. CEVd is around 370 nucleotides in size and belongs to genus *Pospiviroid* of family *Pospiviroidae*. It has a wide host range, including woody species such as ‘Etrog’ citron (*Citrus medica* L.) and herbaceous species such as tomato [28,29,30,31]. “Arizona 861-S-1”, a selection of ‘Etrog’ citron, is generally used for biological indexing purposes and displays some specific syndromes after being infected with citrus viroids [28]. Microarray analysis has revealed that viroid infection could trigger changes in the expression of genes involved in chloroplast function, cell wall structure, peroxidase, and symporter activity in the ‘Etrog’ citron [13]. 

In this study, we employed the CEVd-infected citron system to detect host genome-wide changes using RNA-seq. We analyzed the response of the woody host to the viroid and revealed a large number of genes involved in the defense response, indicating activation of plant immunity following CEVd infection. Our results would help to elucidate the global changes in the expression of the viroid-infected woody host genes and promote the development of effective measurements of viroid diseases in woody plants, thereby contributing to a better understanding of the pathogenesis of viroids.

## 2. Materials and Methods

### 2.1. Preparation of Infectious CEVd RNAs

CEVd were isolated from Meishan No. 9 [*Citrus sinensis* (L.) Osb.] in China. Sequence analysis was performed using Clustal W program and secondary structure was obtained via MFOLD web server and RNAviz program. Total RNA of approximately 0.1 g of citrus symptomatic leaves was extracted using EASYspin Plus Complex Plant RNA Kit (Aidlab Biotech, Beijing, China), according to the manufacturer’s instructions. Based on the sequences of CEVd (371 bp), one-step RT-PCR analysis using PrimeScript^TM^ One Step RT-PCR Kit Ver.2 (Takara, Beijing, China) was conducted to synthesize full-length cDNA of CEVd-dimer using CEVd-specific primers (CEVd-For: 5’-GGAAACCTGGAGGAAGTCGAG-3’ and CEVd-Rev: 5’-CCGGGGATCCCTGAAGGACTT-3’) [32]. The dimeric products, which were amplified by one-step RT-PCR, were ligated overnight at 4°C using the pGEM-T Easy Vector System (Promega, Beijing, China) to obtain pGEM-CEVd-dimers, which were dimeric linker products. All the entire cDNA inserts were transformed into *Escherichia coli DH5α* competent cells. Then, the expected dimeric recombinant plasmids were identified by sequencing and selected out. The plasmids were extracted using Plasmid DNA Mini Kit I (EZNA, Shanghai, China), linearized with the *Spe*I enzyme, and then purified by ethanol. The linearized plasmids containing the full-length dimeric cDNA of CEVd were used as templates for *in vitro* transcription. Infectious CEVd RNAs were generated by *in vitro* transcription with T7 RNA polymerase (Promega, Madison, WI, USA) according to the manufacturer’s instructions.

### 2.2. Inoculation of the Viroid-Free Plants

CEVd RNAs diluted to 500 ng/μL with buffer (100 mM Tris-HCl, 10mM EDTA, pH 7.5) were mechanically inoculated onto viroid-free plants. Each of the five ‘Etrog’ citron (knife cutting for inoculation) or tomato plants (friction used carborundum for inoculation) was inoculated with CEVd RNAs and five other viroid-free plants were used as healthy controls. All plants were stored in a 28–32 °C greenhouse. CEVd systemic infection was verified by one-step RT-PCR as described above. Finally, ‘Etrog’ citron seedlings were inoculated via bark grafting. Seedlings infected with CEVd were treated groups, while viroid-free seedlings were healthy controls. After 3 months of storage in the 28–32 °C greenhouse, the leaves of the citron plants were collected for transcriptome sequencing.

### 2.3. Northern Blot Hybridization

Northern blot hybridization using DIG-labelled CEVd-specific RNA probes was conducted to verify infection activity. DIG Northern Starter Kit Ver.10 was used for northern blot analysis as instructed by the manufacturer (Roche, Mannheim, Germany). Equal amounts of total RNA from CEVd-infected tomato plants and healthy controls were electrophoresed in an acetaldehyde-containing agarose gel. The RNAs in the agarose gel were then transferred to a Hybond N^+^ nylon membrane using a capillary transfer system. Northern blot hybridization was performed at 68 °C for at least 6 h with the CEVd-dimer full length probe. The hybridized probes were immunodetected with anti-digoxigenin-AP, Fab fragments and then visualized with the colorimetric substrates NBT/BCIP.

### 2.4. Library Construction and Transcriptome Sequencing

Three biological replicates were separately used for the treatment group and healthy controls, and each biological replicate included at least five plants to eliminate differences between individual plants. Equal amounts of total RNA from CEVd-infected and mock-inoculated plants were used for RNA sequencing. Approximately 1 µg of RNA per sample was used to prepare RNA samples. The NEBNext® Ultra™ RNA Library Prep Kit (NEB, Ipswich, USA) was used to construct sequencing libraries, and index codes were added to the attribute sequence for each sample. The library preparations were performed by Beijing Novogene Bioinformation Technology Company and then sequenced on an Illumina HiSeq 2500 platform. The raw RNA-seq datasets are available at NCBI (accession no: PRJNA542205).

### 2.5. Identification and Enrichment Analysis of Differentially Expressed Genes (DEGs)

High-quality citron genome was used for mapping, and high mapping scores were conducive to subsequent data analysis [33]. The DESeq R package was used to perform differential expression analysis between the two groups [34]. The DESeq statistical program uses a model based on a negative binomial distribution to determine differential expression in digital gene expression data. The Benjamini and Hochberg methods were used to adjust the resulting *p*-values. The adjusted *p*-value <0.05 genes were designated as differentially expressed.

Gene Ontology (GO) enrichment analysis of DEGs was performed using the GOseq R package [35]. GO terms with corrected *p*-value < 0.05 were considered significantly enriched. The Kyoto Encyclopedia of Genes and Genomes (KEGG) is a database resource for understanding high-level functions and utilities of the biological system such as the cell, the organism and the ecosystem, from molecular-level information, especially large-scale molecular datasets generated by genome sequencing and other high-throughput experimental technologies (http://www.genome.jp/kegg/). The KOBAS software [36] was used to perform statistical enrichment analysis of DEGs in KEGG pathways. KEGG terms with corrected *p*-value < 0.05 were considered to be significantly enriched in DEGs.

### 2.6. Validation of DEGs by RT-qPCR

Some DEGs were randomly selected for quantitative real-time PCR (RT-qPCR) analysis using specific primer pairs to validate our transcriptome data (Table S1). Primer 5.0 was used to design suitable primers for qPCR. Template cDNA was synthesized by means of M-MLV reverse transcriptase and random hexamer primers. A IQ5 real-time PCR detection system (Bio-Rad, CA, USA) was used to perform PCR amplification. The amplification system (20 µL) included diluted cDNA, 10 µL SYBR Green Real-Time PCR Master Mix and 10 µM forward and reverse specific primers. The reaction conditions were as follows: 95 °C for 5 min, and then 40 cycles at 95 °C for 15 s, at 58 °C for 30 s, and at 72 °C for 30 s. Ct (2^−DDCt^) was calculated to determine the relative expression levels of the selected genes [37]. The citron actin gene was selected as internal reference to normalize gene expression levels [38]. Three independent biological replicate assays were performed to reduce the error.

## 3. Results

### 3.1. Infectivity Confirmation of CEVd RNAs

**Figure 1:**
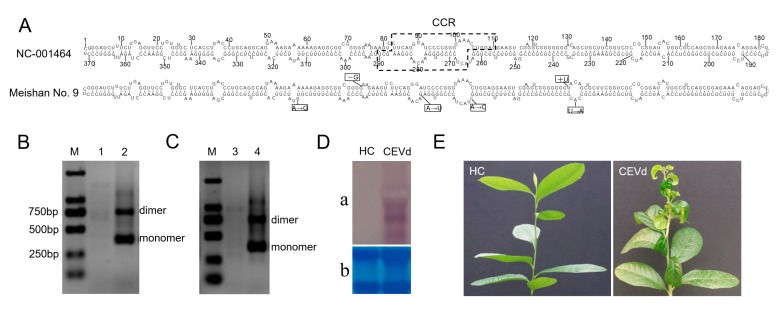
Primary and secondary structures of CEVd variant, and disease symptoms of citron plants infected with the CEVd variant. (**A**) The positions of nucleotides in CEVd variant used in this study that differ from CEVd-Reference (NC_001464). (**B**) One-step RT-PCR products of CEVd-infected citrus materials. (**C**) One-step RT-PCR analysis of CEVd-inoculated citron. (**D**) Northern blot hybridization using specific probes of nucleic acid preparations derived from CEVd-infected tomato leaves and healthy control. (**E**) Different symptoms between citron plants inoculated with the CEVd variant and healthy control. M, BM2000 DNA marker; 1 and 3, viroid-free samples; 2, CEVd-infected sample; 4, ‘Etrog’ citron inoculated with CEVd RNA transcripts; a, Northern blot; b, RNA control; HC, healthy control.

Four nucleotide differences were identified between the CEVd variant used in this study and CEVd-Reference (NC-001464) deposited in NCBI (Figure 1A). We successfully amplified CEVd-dimers using one-step RT-PCR, and the RT-PCR products of approximately 742 bp in size were observed in CEVd-infected samples (Figure 1B). Sequencing analysis showed that the genome sequences of CEVd-dimers were truly 742 bp in length. To verify whether infectious clones of CEVd with the cDNA dimers obtained in this study were effective, the viroid-free plants were inoculated with infectious CEVd dimeric RNAs. Infectivity was assayed by RT-PCR and northern blot analysis of leaves that were prepared from the bioassay plants. The presence of CEVd RNAs in the top fresh leaves of the citron plant was determined by RT-PCR three months after inoculation (Figure 1C). Northern blot analysis of leaf samples from inoculated tomato plants further confirmed that the transcripts of CEVd could systemically infect viroid-free plants (Figure 1D). The appearance of disease symptoms on citron seedlings was observed within four months after inoculation. The ‘Etrog’ citron seedlings that were infected with RNA transcripts showed a severe syndrome compared to the uninfected ‘Etrog’ citron, which was characterized by stunting, leaf curling, and midvein, petiole, and stem necrosis (Figure 1E). Similar results were observed in all five independent plants, confirming that CEVd appears to be a severe variant in citron.

### 3.2. Transcriptome Sequencing and Gene Expression Analysis

**Figure 2:**
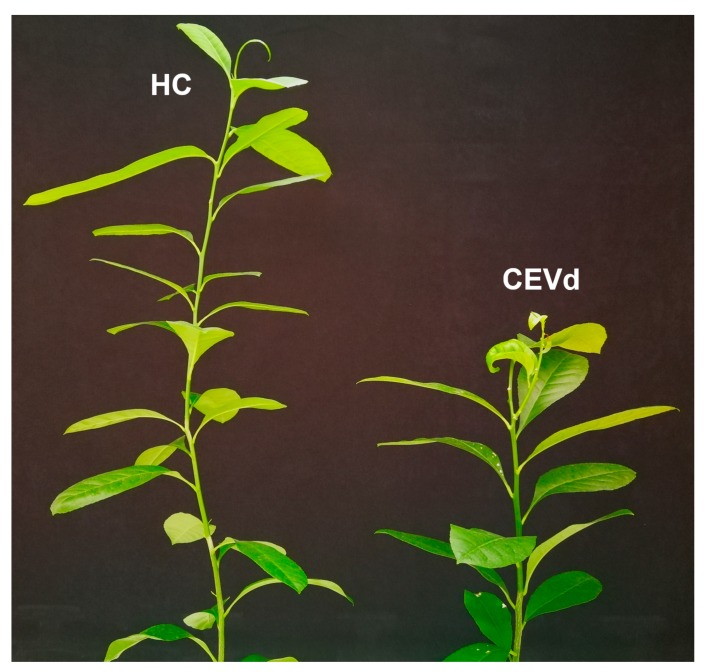
Disease symptoms of citron plants after grafting for 3 months.

To analyze the interaction of CEVd with citron at the transcriptional level, 10 other CEVd-infected citron plants were obtained by bark grafting, and the parietal leaves of the citron plants at 3 months of healthy control and CEVd infection were collected for transcriptome analysis (Figure 2). This was a critical stage in the beginning of CEVd symptoms. We constructed and sequenced RNA-Seq libraries of the mock control and CEVd-infected citron leaves with three biological replicates. RNA-seq yielded 73.26–117.80 million raw reads and retained 67.75–112.71 million clean reads after processing the sequencing data (Table 1). Quality control parameters indicated that the data obtained by RNA-seq were reliable, and gene expression pattern correlation analysis between biological replicate samples indicated high reproducibility of sequencing (Figure S1).

**Figure 3:**
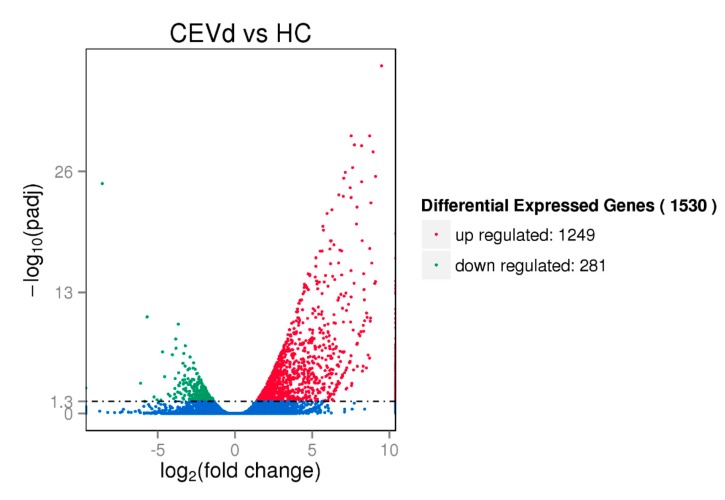
Volcano map of the differential genes. Genes with significant differential expression were indicated by red dots (upregulated) and green dots (downregulated). Genes with no significant differential expression were represented by blue dots.

FPKM (expected number of Fragments Per Kilobase of transcript sequence per Million base pairs sequenced) was used to calculate gene expression levels, and DEGSeq package was used to compare the expression levels of genes identified in different treatments. CEVd infection caused a rich change in gene expression of citron leaves. CEVd induced differential expression of 1530 genes in citron leaves, of which 1249 genes were significantly upregulated, and 281 genes were significantly downregulated (Figure 3, Tables S2 and S3). The heat cluster map shows the DEG expression patterns between CEVd-infected citron plants and healthy controls (Figure S2).

### 3.3. Gene Enrichment Analysis

GO enrichment analysis was performed to analyze the function of DEGs in response to CEVd infection, and the results provided an overview of statistically significant and relevant GO terms. GO terms with a corrected *p* value < 0.05 were considered to be enriched. Upregulated DEGs were mainly enriched in transcription, RNA biosynthesis, chitin metabolism and protein kinase activity in citron plants (Table 2), while downregulated DEGs were mainly enriched in the terms related to auxin reactions and cell wall (Table 3). The GO terms related to transcription on biological process and the terms that were related to chitinase activity on molecular function were the most significantly upregulated, whereas the terms related to plant hormone on biological process and the terms related to cell wall on the cellular component were the most significantly downregulated.

KEGG enrichment analysis was performed to identify major metabolic and signal transduction pathways that might be disrupted during CEVd infection. The results showed that four upregulated enrichment KEGG pathways were glutathione metabolism, plant-pathogen interaction, secondary metabolite biosynthesis, and amino sugar and nucleotide sugar metabolism, whereas the downregulated enrichment KEGG pathways were phytohormone signaling, phenylpropanoid biosynthesis and phenylalanine metabolic pathways (Table 4 and Table 5). Especially, 52 genes involved in secondary metabolite biosynthesis including flavonoid biosynthesis were found to be significantly upregulated against the CEVd infection in citron plants.

### 3.4. CEVd Infection Induces Expression of Many Genes That Are Related to Basal Defense Responses

Viroid infections can cause disease symptoms, and plants have evolved basic immunity to limit diseases, which are activated at the site of infection and then spread when the plants are attacked by the pathogens [39,40,41]. A central component of signal transduction in the PTI and ETI pathways is the mitogen-activated protein kinase (MAPK) cascade, which activates symptom-related genes including WRKY transcription factors. Our RNA-seq results showed upregulation of genes encoding MAPKs (mitogen-activated protein kinase/mitogen-activated protein kinase kinase/mitogen-activated protein kinase kinase kinase) and WRKY transcription factors. Receptor-like kinase (RLK), a membrane-localized protein, could identify pathogen avirulence determinants, and the LRR-RLK genes were involved in plant innate immunity. CEVd infection induced the expression of the LRR genes in the citron plants. The genes encoding cyclic nucleotide-gated channel (CNGC), respiratory burst oxidase (Rboh), calcium-binding protein CML, heat shock cognate 70 kDa protein, pathogenesis-related protein, and disease resistance protein were also upregulated in CEVd-infected citron plants (Table S4). These genes might play a key role in regulating the innate immune response and revealed potential regulatory elements in citron in response to CEVd infection.

### 3.5. CEVd Infection Impacts Plant Hormone Signaling

Plant hormones regulate plant growth and development, and infection by viruses and viroids can affect a variety of plant hormone signaling pathways and lead to disease symptoms in the infected plants [12,42]. KEGG enrichment analysis was performed to identify DEGs involved in the phytohormone signaling pathway. The result showed that citron plants infected with CEVd had many DEGs associated with phytohormone signal transduction compared to mock-inoculated plants (Table S5). Some DEGs participated in the auxin (IAA), jasmonic acid (JA), brassinosteroid (BR), and gibberellin (GA) signal transduction pathways and downregulation of genes involved in the IAA signal transduction pathway may be associated with the appearance of disease symptoms such as stunting on CEVd-infected citron plants. The DEGs involved in JA, BR, and GA signal transduction pathways were upregulated in CEVd-infected citron leaves.

### 3.6. RNA Silencing Responses to CEVd Infection

**Figure 4:**
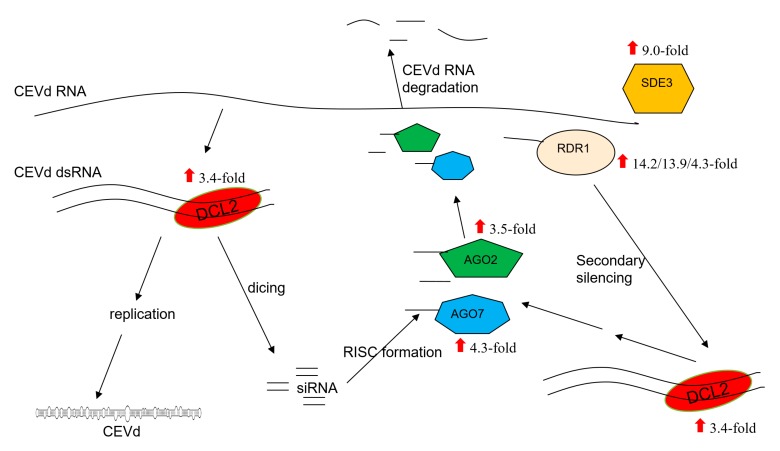
Schematic depiction of the antiviral RNA silencing pathway with differentially expressed genes and their mRNA fold change based on RNA-sequencing (RNA-seq) analysis are shown. DCL2, DICER-LIKE 2; AGO2, ARGONAUTE 2; AGO7, ARGONAUTE 7; RDR1, RNA-DEPENDENT RNA POLYMERASE 1; SDE3, SILENCING DEFECTIVE 3; RISC, RNA-induced silencing complex.

RNA silencing plays a major role in plant defense against RNA and viroid infections [43]. RNA silencing not only participates in plant antiviral responses, but also directly induces viroid diseases in plants [44,45]. Therefore, we screened DEGs related to the RNA silencing pathway, including genes encoding DCLs, AGOs, and RNA-dependent RNA polymerases (RDRs). We found that the expression of *DCL2* (Cm146130) was highly upregulated (3.4-fold) in CEVd-infected citrons. Expression of the citron *AGO2* gene (Cm196770) was 3.5-fold upregulated and the gene expressing *AGO7* (Cm010810) was upregulated 4.3-fold in CEVd-infected citrons. Especially, we also found that the expression of three citron *RDR1* gene dramatically increased in CEVd-infected citron plants (Novel01970 by 14.2-fold, Novel01971 by13.9-fold, and Cm225400 by 4.3-fold). In addition, the expression of the PTGS-related gene *SILENCING DEFECTIVE 3* (*SDE3*) (Cm260670) was also upregulated in CEVd-infected citron plants (Figure 4, Table S6).

### 3.7. Validation of RNA-seq Results by RT-qPCR

**Figure 5:**
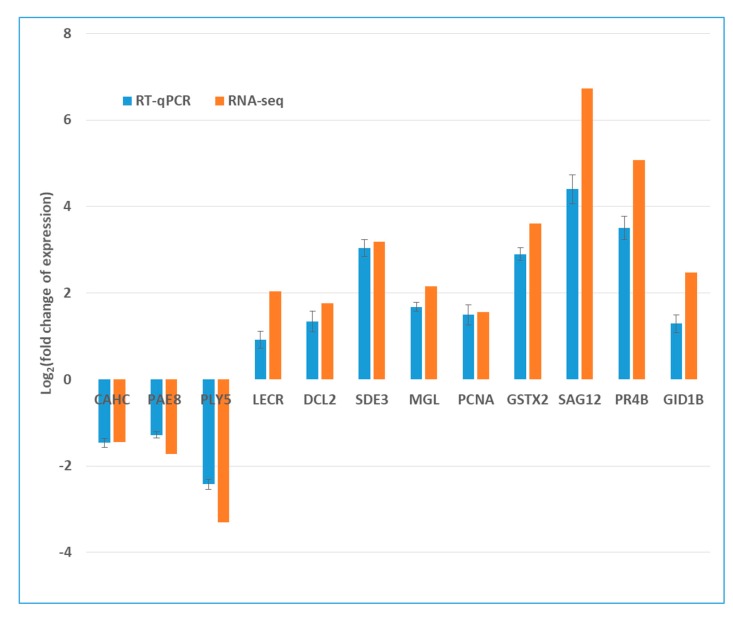
Validation of RNA-seq results by quantitative real-time PCR (RT-qPCR). Expression patterns of 12 representative genes as determined by RT-qPCR and RNA-seq. Normalization for RT-qPCR was performed using expression of the actin gene as an internal reference. CAHC: Carbonic anhydrase, chloroplastic; PAE8: Pectin acetylesterase 8; PLY5: Pectate lyase 5; LECR: Lectin-related protein; DCL2: Endoribonuclease Dicer homolog 2; SDE3: Silencing defective 3; MGL: Methionine gamma-lyase; PCNA: Proliferating cell nuclear antigen; GSTX2: Glutathione S-transferase; SAG12: Senescence-specific cysteine protease SAG12; PR4B: Pathogenesis-related protein PR-4B; GID1B: Gibberellin receptor GID1B.

To validate the RNA-seq results, 12 DEGs related to responses to CEVd infection were randomly selected and their expression levels were analyzed by RT-qPCR using specific designed gene-specific primers. The expression changes of these genes were similar to those of the RNA-seq data, indicating that the RNA-seq results were reliable (Figure 5, Table S7).

## 4. Discussion

Citrus is one of the most important fruits in the world, and the research and control of citrus diseases have important economic significance. Specific strains of CEVd can cause pronounced symptoms on sensitive citrus species and can be rapidly spread in commercial orchards by mechanical means. Early studies compared the differences in tomato transcriptomes induced by PSTVd [14], and two studies examined changes of host gene expression in different tomato cultivars following CEVd [13] or PSTVd infection [12]. These studies are all evaluating the response of herbaceous hosts to viroid infection. Here, we used RNA-seq analysis to analyze changes of gene expression associated with CEVd infection in a woody host. A high quality citron genome reported recently was used for mapping analysis and higher mapping rates were conducive to obtain ideal results [33]. The results highlighted the transcriptomic changes in the leaves of citron plants caused by CEVd infection, which showed that CEVd infection affected the expression of several important genes that are involved in the basic defense response, phytohormone signal transduction, RNA silencing pathway, and some other pathways such as secondary production synthesis pathways.

Plants have an innate immune system called the basic defense response that identifies invading pathogens and initiates effective defense [46]. As observed in this study, plants convert the perception of pathogen invasion into signal cascades containing CNGCs, which increase Ca^2+^ concentrations and activate CMLs [47]. *Rboh* genes were upregulated by CEVd in citron leaves, and Rboh was associated with ROS production in *Arabidopsis* [48]. Expression of the LRR receptor-like kinase FLS2 was also upregulated by CEVd infection, which combines with BAK1 to form a complex [46,49]. The signal was then passed from FLS2 to MEKK1, which had been reported to activate the MAPK cascade in *Arabidopsis* [46,50]. Pathogen infection usually results in transcriptional changes in the host. A hypersensitive response (HR) leads to significant changes in gene expression patterns and causes large increases of many different proteins, which include members of the PR and disease resistance protein families. We observed a significant upregulation of PR gene expression in CEVd-infected plants, which agreed with the findings of previous studies. *Apple stem groove virus* (ASGV) infection induces the upregulation of the gene encoding the PR protein in apple [51], and *tomato spotted wilt virus* (TSWV) and CEVd infections induce PR expression in tomato [52]. CEVd infection also induced the upregulation of genes encoding disease resistance-related proteins in citron plants, suggesting that CEVd infection triggers a plant immune response. HSP family homologs are significantly induced in many plants infected with RNA viruses and viroids [44,53]. It has been suggested that HSP is involved in the regulation of host defense responses in hosts infected by RNA virus [54]. In this study, HSP70 transcripts were found to be induced to higher levels in CEVd-infected citron, suggesting that HSP70 played an important role in the CEVd infection cycle. Transcription factors (TFs) often play an important role against abiotic and biotic stresses in plants [55]. The genes of major TF families (WRKYs, MYBs, and ERFs) were significantly changed in CEVd-infected citron plants, consistent with the findings of previous studies involving PSTVd-infected potatoes [56]. CEVd does not encode any proteins, and how the viroid activates ETI is an interesting and important question. It has been reported that protein kinase viroid-induced (PKV) genes are involved in the development of symptoms during infection with viroids [57]. Numerous PKV genes were upregulated in this study and they might be associated with the actiovation of ETI in citron plants.

Many studies have shown that phytohormones are regulators of many important metabolic pathways associated with abiotic/biotic stress responses and plant growth and development and play important roles in the life cycle of plants [58,59]. Infection by pathogens such as viruses and viroids often alters plant hormone accumulation and signaling, resulting in physiological destruction of the host cells and plant developmental disorders [12,42,57,60,61,62]. In our study, the expression of multiple genes associated with the plant hormone signal transduction was altered, and some important plant hormone signaling pathways were affected after CEVd infection. We found that CEVd infection induced the upregulation of genes encoding components of the JA signal transduction pathway, and JA may be involved in the interaction between viroids and plants. Some genes involved in GA and BR signal transduction pathways were also upregulated, whereas many genes involved with IAA were downregulated. These results suggest that CEVd infection simultaneously alters several plant hormone signaling pathways, and the relationship between viroids and plant hormones is complex. Recently, salicylic acid (SA) has attracted attention in improving plant basic resistance against viroids [52]. However, no changes in gene expression involved in the SA signal transduction pathway were observed in this study. Similar results were also observed in tomato plants after PSTVd infection, which showed alterations in transcript levels of several genes related to GA and BR signaling, but none of other genes involved in the SA-dependent pathway [12].

RNA silencing plays a major role in plant defense mechanisms against RNA viruses and viroids because their genomes can be directly targeted by DCL proteins and RNA-induced silencing complexes (RISCs) [45,63]. In this study, the genes encoding key components such as DCL2, RDR1, AGO2, and AGO7 of the gene silencing pathway were upregulated in CEVd-infected citron plants. RNA silencing begins with the formation of double-stranded RNA (dsRNA) molecules, which are substrates for DCL proteins [64]. There are four DCL proteins that are involved in RNA silencing in *Arabidopsis*. DCL2 and DCL4 have overlapping functions in antiviral RNA silencing defense, and DCL2 is required to generate secondary small interfering RNA (siRNA) [65,66]. The expression of the *DCL2* gene was upregulated in CEVd-infected citron, indicating that DCL2 may play a major role in antiviral defense in woody plants. The AGO proteins in plants are an important component of RISCs. AGO proteins have antiviral functions, and AGO2 has a wide range of effects in antiviral silencing [67,68,69,70]. AGO1 and AGO7 have also been shown to play a role in plant antiviral silencing pathways in *Arabidopsis thaliana* [71]. The genes encoding AGO2 and AGO7 were significantly upregulated in CEVd-infected citron, whereas the genes encoding AGO1 and other AGO proteins showed no significant changes. The results indicate that AGO7 possibly acts synergistically with AGO2 to suppress CEVd infection to a greater degree than the other AGO proteins in citron plants. RDR1 plays an important role in PTGS immune response [65,72,73,74]. This study found that CEVd infection induced the upregulated expression of three RDR1 genes in citron plants, consistent with previous findings in PSTVd-infected tomato [75] and HSVd-infected cucumber [22], indicating that RDR1 is involved in viroid-host interactions. Early studies have reported that RNA silencing is an important mediator of host-viroid interactions [76], and RDR1 is one of the major components involved in the RNA silencing pathway [77]. It can lead to basal resistance to some viruses by producing virus-derived siRNA [65,73,78,79]. In *Arabidopsis*, RDR1 can also confer broad-spectrum antiviral activity by generating viral-activated siRNA (vasiRNA) resulting in extensive silencing of the host genes [80]. These results indicate that RDR1 may be crucial in antiviral and viroid resistance by silencing the RNA of viruses and viroids as well as host immune-related genes. In addition to RDR1, RDR2 and RDR6 are also reported to be involved in viroid-host interactions [73]. No evidence of transcriptional changes in RDR2 or RDR6 was found in this study, but the differences might be due to various viroids and plants. In addition, the upregulated *SDE3* gene found in this study is reported to encode RNA helicases in *Arabidopsis* and is also important for PTGS [81]. Notably, RT-qPCR results further showed that the expression levels of *DCL2* and *SDE3* was upregulated in response to CEVd infection and they might play important roles in the interaction between CEVd and citron plants.

In addition, we analyzed the GO terminology of DEGs in CEVd-infected citron plants, and found that some enriched GO terms are related to chitinase activity and cell wall, which coincides with the malformation symptoms of citron leaves. Early studies have reported that CEVd infection could change cell wall thickness of the epidermal cells, disorganize spongy and palisade mesophyll, and induce callose deposits in citron plants with the help of confocal laser scanning microscopy, which was confirmed by the enriched GO terms related to cell wall in the present study [13]. KEGG analysis also revealed that CEVd infection caused the differential expression of numerous genes involved in secondary metabolite biosynthesis. Phenylalanine ammonia lyase (PALY) is an important enzyme involved in the biosynthesis of secondary metabolites such as flavonoids [82]. In this study, the expression of the PALY gene increased in the CEVd-infected plants. Flavonoids are components of many phenolic secondary metabolites with a variety of biological functions such as defense against biotic stresses [83]. The observed upregulation of genes in the flavonoid biosynthetic pathway suggested that CEVd infection stimulated the accumulation of defense substances in citron.

In summary, we have studied the transcriptional profile of citron leaves infected with CEVd and healthy controls. Compared to uninfected leaves, CEVd infection triggers basic defense responses, destroys plant hormone homeostasis, induces the expression of key genes involved in RNA silencing, and affects cell wall and secondary metabolism in citron plants. Our findings will help elucidate the response mechanisms of woody plants against viroid infections and facilitate in the development of strategies to combat viroid diseases in fruit trees.

## Figures and Tables

**Table 1:** The quality of sequencing data.

Sample Name	Raw Reads	Clean Reads	Clean Bases	Q20 (%)	Q 30(%)	GC Content (%)
HC1	86,594,558	83,854,342	12.45G	97.66	93.99	44.75
HC2	73,264,886	68,562,642	10.16G	97.51	94.13	48.73
HC3	95,947,986	89,863,580	13.24G	97.68	94.31	44.36
CEV1	117,995,510	113,662,956	16.91G	97.72	94.10	44.35
CEV2	107,136,484	104,075,096	15.50G	97.87	94.72	42.76
CEV3	80,867,964	75,965,424	11.32G	97.73	94.39	42.65

**Table 2:** Enriched GO terms for the upregulated DEGs in CEVd-infected citron plants.

GO Accession	Description	Term Type	Corrected *p*-Value
GO:0006351	transcription, DNA-templated	BP	8.13 × 10^−5^
GO:0097659	nucleic acid-templated transcription	BP	8.13 × 10^−5^
GO:0032774	RNA biosynthetic process	BP	8.13 × 10^−5^
GO:0019219	regulation of nucleobase-containing compound metabolic process	BP	8.13 × 10^−5^
GO:0006355	regulation of transcription, DNA-templated	BP	8.13 × 10^−5^
GO:1903506	regulation of nucleic acid-templated transcription	BP	8.13 × 10^−5^
GO:0051252	regulation of RNA metabolic process	BP	8.13 × 10^−5^
GO:2001141	regulation of RNA biosynthetic process	BP	8.13× 10^−5^
GO:0006030	chitin metabolic process	BP	8.13 × 10^−5^
GO:1901071	glucosamine-containing compound metabolic process	BP	8.13 × 10^−5^
GO:0006032	chitin catabolic process	BP	8.13 × 10^−5^
GO:0046348	amino sugar catabolic process	BP	8.13 × 10^−5^
GO:1901072	glucosamine-containing compound catabolic process	BP	8.13 × 10^−5^
GO:0016998	cell wall macromolecule catabolic process	BP	0.000133
GO:0006026	aminoglycan catabolic process	BP	0.000133
GO:2000112	regulation of cellular macromolecule biosynthetic process	BP	0.001066
GO:0006040	amino sugar metabolic process	BP	0.001132
GO:0010556	regulation of macromolecule biosynthetic process	BP	0.001132
GO:0031326	regulation of cellular biosynthetic process	BP	0.001132
GO:0009889	regulation of biosynthetic process	BP	0.001235
GO:0051171	regulation of nitrogen compound metabolic process	BP	0.001391
GO:0034654	nucleobase-containing compound biosynthetic process	BP	0.002117
GO:0044036	cell wall macromolecule metabolic process	BP	0.004343
GO:0010468	regulation of gene expression	BP	0.004949
GO:0080090	regulation of primary metabolic process	BP	0.006186
GO:0031323	regulation of cellular metabolic process	BP	0.00663
GO:0060255	regulation of macromolecule metabolic process	BP	0.009714
GO:0018130	heterocycle biosynthetic process	BP	0.013439
GO:1901362	organic cyclic compound biosynthetic process	BP	0.013439
GO:0006022	aminoglycan metabolic process	BP	0.014413
GO:0019438	aromatic compound biosynthetic process	BP	0.015638
GO:0019222	regulation of metabolic process	BP	0.02637
GO:0043207	response to external biotic stimulus	BP	0.033447
GO:0051707	response to other organism	BP	0.033447
GO:0006468	protein phosphorylation	BP	0.040882
GO:0004568	chitinase activity	MF	8.13 × 10^−5^
GO:0001071	nucleic acid binding transcription factor activity	MF	0.000204
GO:0003700	transcription factor activity, sequence-specific DNA binding	MF	0.000204
GO:0004674	protein serine/threonine kinase activity	MF	0.000761
GO:0004672	protein kinase activity	MF	0.014413
GO:0008061	chitin binding	MF	0.0321
GO:0020037	heme binding	MF	0.033855

BP, biological process; CC, cellular component; MF, molecular function.

**Table 3:** Enriched GO terms for the downregulated DEGs in CEVd-infected citron plants.

GO Accession	Description	Term Type	Corrected *p*-Value
GO:0009733	response to auxin	BP	1.70 × 10^−6^
GO:0009725	response to hormone	BP	1.07 × 10^−5^
GO:0009719	response to endogenous stimulus	BP	1.07 × 10^−5^
GO:0010033	response to organic substance	BP	3.29 × 10^−5^
GO:0045229	external encapsulating structure organization	BP	0.002848
GO:0044264	cellular polysaccharide metabolic process	BP	0.004845
GO:0009832	plant-type cell wall biogenesis	BP	0.004996
GO:0010215	cellulose microfibril organization	BP	0.004996
GO:0030198	extracellular matrix organization	BP	0.004996
GO:0043062	extracellular structure organization	BP	0.004996
GO:0070726	cell wall assembly	BP	0.004996
GO:0071668	plant-type cell wall assembly	BP	0.004996
GO:0042221	response to chemical	BP	0.007365
GO:0071555	cell wall organization	BP	0.007365
GO:0006073	cellular glucan metabolic process	BP	0.007365
GO:0044042	glucan metabolic process	BP	0.007365
GO:0005976	polysaccharide metabolic process	BP	0.008838
GO:0016049	cell growth	BP	0.013705
GO:0044262	cellular carbohydrate metabolic process	BP	0.025726
GO:0009664	plant-type cell wall organization	BP	0.027923
GO:0071669	plant-type cell wall organization or biogenesis	BP	0.027923
GO:0042546	cell wall biogenesis	BP	0.036742
GO:0071554	cell wall organization or biogenesis	BP	0.037842
GO:0040007	growth	BP	0.038351
GO:0005618	cell wall	CC	0.004996
GO:0031225	anchored component of membrane	CC	0.007945
GO:0030312	external encapsulating structure	CC	0.018609
GO:0005507	copper ion binding	MF	9.43 × 10^−5^

BP, biological process; CC, cellular component; MF, molecular function.

**Table 4:** KEGG pathway enrichment of DEGs from CEVd-infected citron plants.

Term	ID	Input Number	Background Number	Corrected *p*-Value
**Up-Regulated Kegg Pathways (Corrected *p*-Value<0.05)**				
Glutathione metabolism	cit00480	18	95	6.45 × 10^−6^
Plant-pathogen interaction	cit04626	18	208	0.044203
Biosynthesis of secondary metabolites	cit01110	52	930	0.047811
Amino sugar and nucleotide sugar metabolism	cit00520	12	120	0.047811
**Down-Regulated Kegg Pathways (Corrected *p*-Value < 0.05)**				
Plant hormone signal transduction	cit04075	10	217	0.000251
Phenylpropanoid biosynthesis	cit00940	7	159	0.004052
Phenylalanine metabolism	cit00360	5	115	0.022122

**Table 5:** Effect of the genes involved in plant-pathogen interactions in CEVd-infected citron plants.

Gene ID	log_2_ Fold Change	*p*-adjusted	Function
Cm116590	1.5929	0.048035	DRL27: Disease resistance protein At4g27190
Cm029390	6.1457	2.21 × 10^−18^	RBOHD: Respiratory burst oxidase homolog protein D
Cm070920	1.6338	0.020128	EFTM: Elongation factor Tu, mitochondrial
Cm216070	Inf *	5.50 × 10^−7^	CML8: Calmodulin-like protein 8
Cm056030	6.7263	3.39 × 10^−24^	CML19: Putative calcium-binding protein CML19
Cm113060	2.7079	4.10 × 10^−6^	CML27: Probable calcium-binding protein CML27
Cm128110	5.2652	0.00012824	CML31: Probable calcium-binding protein CML31
Cm040570	3.4838	1.15 × 10^−7^	CML44: Probable calcium-binding protein CML44
Cm241600	3.0724	4.07 × 10^−7^	ALLB3: Calcium-binding allergen Bet v 3
Cm050430	2.513	0.00030948	ALL8: Calcium-binding allergen Ole e 8
Novel01586	–2.1223	0.036209	PREDICTED: calcium-dependent protein kinase 9-like
Cm106570	4.4746	2.62 × 10^−6^	CNG13: Putative cyclic nucleotide-gated ion channel 13
Cm226290	2.8711	0.00020247	WRK33: Probable WRKY transcription factor 33
Cm011420	3.1569	9.55 × 10^−8^	TIF9: Protein TIFY 9
Cm220810	3.0776	3.14 × 10^−6^	TI10A: Protein TIFY 10A
Cm157630	4.425	3.17 × 10^−8^	TI10A: Protein TIFY 10A
Cm199700	3.0945	0.0029031	CERK1: Chitin elicitor receptor kinase 1
Cm178700	4.3046	0.030672	RIN4: RPM1-interacting protein 4
Cm178710	2.4288	0.0032793	RIN4: RPM1-interacting protein 4

* Inf, Infinite (The denominator is zero.).

## Supplementary Materials

The following are available online at https://www.mdpi.com/1999-4915/11/5/453/s1, Figure S1: Pearson correlation among samples. An *R*^2^ value close to 1 indicates a high degree of correlation among samples. Figure S2: Cluster analysis of differentially expressed genes between CEVd infected citron plants and healthy control. The values of log_10_ (FPKM + 1) are normalized (scale number) and clustered; red, high expression genes; blue, low expression genes; the red and blue colors represent the values of log_10_ (FPKM + 1) from large to small. Table S1: Oligonucleotide primers used in RT-qPCR analysis. Table S2: Upregulated DEGs in citron plants against CEVd infection. Table S3: Downregulated DEGs in citron plants against CEVd infection. Table S4: Effect of CEVd infection on the expression of citron genes in the basal defense response. Table S5: Effect of the genes of plant hormone signal transduction in CEVd-infected citron. Table S6: Effect of CEVd infection on the expression of citron genes in RNA silencing pathway. Table S7: Validation of RNA-seq results by RT-qPCR.
