# Supplementary figures and images for: Global Transcriptomic Analysis Reveals Insights into the Response of ‘Etrog’ Citron (Citrus medica L.) to Citrus Exocortis Viroid Infection
Source: Viruses. 2019 May 17;11(5):453. doi: 10.3390/v11050453 (PMC6563217; doi:10.3390/v11050453)

Pearson correlation between samples

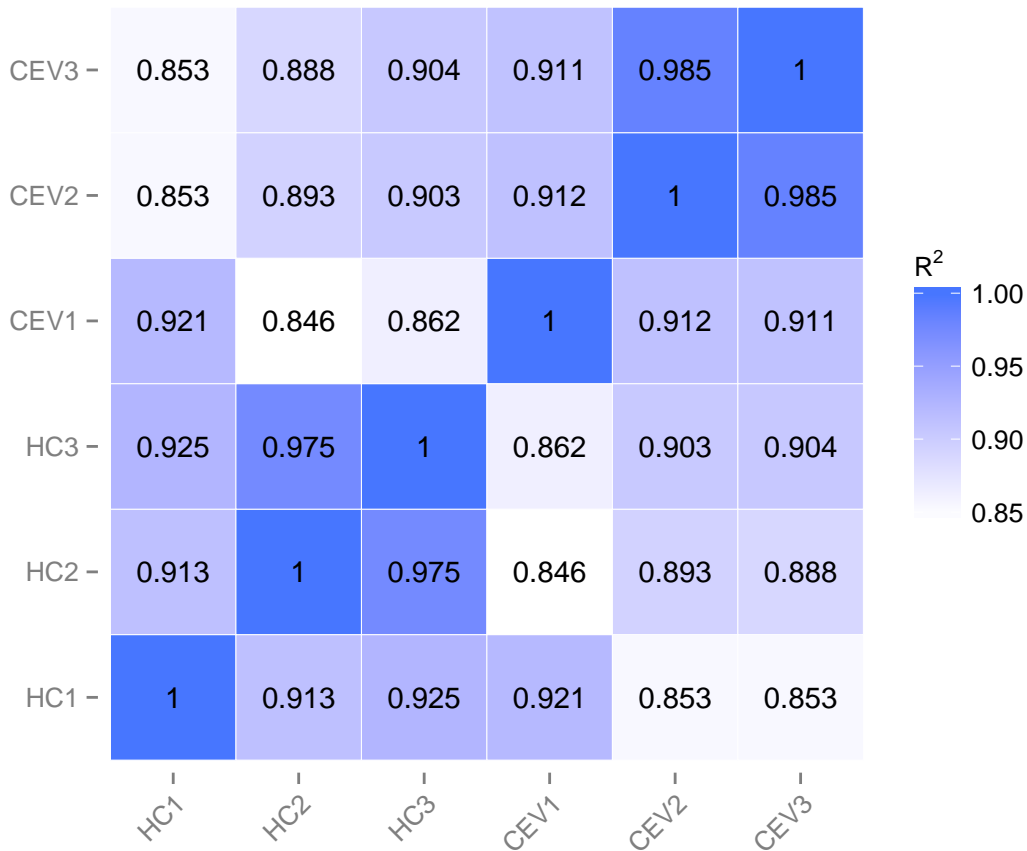

Supplement: Supplementary file 1 [file viruses-11-00453-s001.zip › viruses-485757-supplementary/Supplementary files/Figure S1.pdf]

Cluster analysis of differentially expressed genes

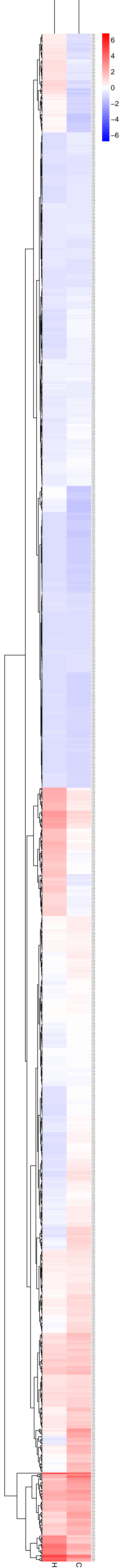

Supplement: Supplementary file 1 [file viruses-11-00453-s001.zip › viruses-485757-supplementary/Supplementary files/Figure S2.pdf]
