# Supplementary material for: Global Transcriptomic Analysis Reveals Insights into the Response of ‘Etrog’ Citron (Citrus medica L.) to Citrus Exocortis Viroid Infection
Source: Viruses. 2019 May 17;11(5):453. doi: 10.3390/v11050453 (PMC6563217; doi:10.3390/v11050453)
Supplement: Supplementary file 1 [file viruses-11-00453-s001.zip › viruses-485757-supplementary/Supplementary files/Table S1.doc]

Table S1. Oligonucleotide primers used in RT-qPCR analysis.

| Gene | Gene ID | F (from 5’ to 3’) | R (from 5’ to 3’) | Efficiency | R^2^ |
| --- | --- | --- | --- | --- | --- |
| CAHC | Cm141580 | AGGGCTTCATTCACTTCA | GAGCAGGCAAATACCATAT | 106.30% | 0.951 |
| PAE8 | Cm246570 | TCTTCCCTGTGGGTACTAAA | CAGTCCTGGGCTCAATCT | 93.20% | 0.921 |
| PLY5 | Cm020020 | CACAGCGATACCTACACCC | GCATACATTTCCCATTGAGT | 93.50% | 0.993 |
| LECR | Cm276170 | GGATGGACGGAAATACTCTA | GGCAGTGGGAATGTTGTG | 92.90% | 0.964 |
| DCL2 | Cm146130 | ATGTCTAATGCTCGTGTTG | ATACCGTTCTTCATAGTGC | 91.60% | 0.999 |
| SDE3 | Cm260670 | ACTGTCAGATCAACCGTCAA | GGATTCCCTACAATAATAAGC | 90.00% | 0.998 |
| MGL | Cm125010 | ATAAGGGAGTTACTGTCGTG | AAGGTCCATCATAGAGTTCA | 100.90% | 0.947 |
| PCNA | Cm099990 | TTCATTCACAAGGGCTAC | AAACTTGAGGCTTGGTCT | 99.60% | 0.98 |
| GSTX2 | Cm261020 | GCAAGAGGCTAAACCAAC | TAGCGAATGAGAAACAGAAA | 96.70% | 0.995 |
| SAG12 | Cm310000 | CAAGGACGACCTGGAGAA | TTGCCGTGATGAAGATGA | 90.10% | 0.987 |
| PR4B | Cm240620 | CTCTACCACCCTGAGCAA | ACCACTGTTTGTAACCCTTA | 98.20% | 0.924 |
| GID1B | Cm240350 | CCGATGGAGAAGATAGAG | CTCAGCCCTTCAACATAA | 104.30% | 0.998 |
