# Supplementary material for: Global Transcriptomic Analysis Reveals Insights into the Response of ‘Etrog’ Citron (Citrus medica L.) to Citrus Exocortis Viroid Infection
Source: Viruses. 2019 May 17;11(5):453. doi: 10.3390/v11050453 (PMC6563217; doi:10.3390/v11050453)
Supplement: Supplementary file 1 [file viruses-11-00453-s001.zip › viruses-485757-supplementary/Supplementary files/Table S4.doc]

Table S4. Effect of CEVd infection on the expression of citron genes in the basal defense response.

| Gene ID | log_2_FoldChange | *p*-adjusted | Function |
| --- | --- | --- | --- |
| Cm169910 | 6.0185 | 2.15E-14 | PTI5: Pathogenesis-related genes transcriptional activator PTI5 |
| Cm194910 | 2.9193 | 0.02886 | PRS2: Pathogenesis-related protein STH-2 |
| Cm240620 | 5.0718 | 8.68E-07 | PR4B: Pathogenesis-related protein PR-4B |
| Cm016300 | 1.684 | 0.013995 | R13L4: Disease resistance RPP13-like protein 4 |
| Cm051590 | -2.8844 | 0.012907 | DR206: Disease resistance response protein 206 |
| Cm116590 | 1.5929 | 0.048035 | DRL27: Disease resistance protein At4g27190 |
| Cm118580 | 4.1283 | 5.74E-11 | RGA2: Disease resistance protein RGA2 |
| Cm246800 | 1.9474 | 0.024089 | RPS4C: Disease resistance protein RPS4 |
| Cm310720 | 3.9813 | 1.27E-11 | HSPR2: Nematode resistance protein-like HSPRO2 |
| Novel00331 | 4.7266 | 0.000255 | PREDICTED: putative disease resistance RPP13-like protein 1-like |
| Novel00579 | 2.1229 | 0.000716 | PREDICTED: putative disease resistance protein At3g14460-like |
| Novel01121 | 1.8205 | 0.031618 | PREDICTED: putative disease resistance protein At4g11170-like |
| Novel01213 | 3.0279 | 8.61E-06 | PREDICTED: putative disease resistance protein RGA3-like |
| Novel01854 | 2.8703 | 0.021672 | PREDICTED: probable disease resistance protein RF9-like |
| Cm057230 | 4.7923 | 0.000313 | CNGC1: Cyclic nucleotide-gated ion channel 1 |
| Cm057260 | 4.4831 | 6.07E-06 | CNGC1: Cyclic nucleotide-gated ion channel 1 |
| Cm106570 | 4.4746 | 2.62E-06 | CNG13: Putative cyclic nucleotide-gated ion channel 13 |
| Cm029390 | 6.1457 | 2.21E-18 | RBOHD: Respiratory burst oxidase homolog protein D |
| Cm056030 | 6.7263 | 3.39E-24 | CML19：Putative calcium-binding protein CML19 |
| Cm040570 | 3.4838 | 1.15E-07 | CML44：Probable calcium-binding protein CML44 |
| Cm113060 | 2.7079 | 4.10E-06 | CML27：Probable calcium-binding protein CML27 |
| Cm128110 | 5.2652 | 0.00012824 | CML31：Probable calcium-binding protein CML31 |
| Cm027010 | 7.2445 | 1.22E-05 | WRK41: Probable WRKY transcription factor 41 |
| Cm033640 | 2.2933 | 0.008969 | WRK23: Probable WRKY transcription factor 23 |
| Cm051960 | 5.1663 | 9.12E-12 | WRK41: Probable WRKY transcription factor 41 |
| Cm058090 | 1.9001 | 0.00543 | WRK40: Probable WRKY transcription factor 40 |
| Cm086930 | 2.8618 | 4.75E-06 | WRK50: Probable WRKY transcription factor 50 |
| Cm101440 | 4.6343 | 0.03382 | WRK28: Probable WRKY transcription factor 28 |
| Cm106450 | 3.669 | 2.09E-10 | WRK30: Probable WRKY transcription factor 30 |
| Cm172370 | 7.0467 | 5.41E-26 | WRK40: Probable WRKY transcription factor 40 |
| Cm174310 | 2.4172 | 0.000332 | WRK75: Probable WRKY transcription factor 75 |
| Cm199430 | 2.2599 | 0.000776 | WRK31: Probable WRKY transcription factor 31 |
| Cm199810 | 4.6701 | 3.43E-14 | WRKY6: WRKY transcription factor 6 |
| Cm226290 | 2.8711 | 0.000202 | WRK33: Probable WRKY transcription factor 33 |
| Novel00794 | 2.4717 | 0.011104 | PREDICTED: probable WRKY transcription factor 49-like |
| Cm127550 | 6.8591 | 1.64E-13 | FLS2: LRR receptor-like serine/threonine-protein kinase FLS2 |
| Novel01787 | 7.1964 | 0.002381 | PREDICTED: LRR receptor-like serine/threonine-protein kinase FLS2-like |
| Cm148130 | 6.2257 | 1.69E-05 | YODA: Mitogen-activated protein kinase kinase kinase YODA |
| Cm299150 | 4.438 | 0.020772 | YODA: Mitogen-activated protein kinase kinase kinase YODA |
| Cm049290 | 1.7969 | 0.009444 | MKKA: Mitogen-activated protein kinase kinase kinase A |
| Cm199450 | 3.01 | 7.03E-07 | MKKA: Mitogen-activated protein kinase kinase kinase A |
| Cm080230 | 2.1059 | 0.001366 | NPK1: Mitogen-activated protein kinase kinase kinase NPK1 |
| Cm142350 | 2.594 | 1.40E-05 | M2K9: Mitogen-activated protein kinase kinase 9 |
| Cm182070 | 1.6279 | 0.019013 | MPK3: Mitogen-activated protein kinase 3 |
| Cm057630 | 5.7813 | 0.000657 | HSP7C: Heat shock cognate 70 kDa protein |
| Cm209370 | 2.461 | 0.000197 | HSP7C: Heat shock cognate 70 kDa protein |
| Cm209380 | 1.9599 | 0.004716 | HSP7C: Heat shock cognate 70 kDa protein |
| Cm209390 | 2.995 | 0.006154 | HSP7C: Heat shock cognate 70 kDa protein |
| Cm209400 | 4.7362 | 2.88E-07 | HSP7C: Heat shock cognate 70 kDa protein |
| Cm318430 | 5.4443 | 7.55E-08 | HSP7C: Heat shock cognate 70 kDa protein |
