# Supplementary material for: Global Transcriptomic Analysis Reveals Insights into the Response of ‘Etrog’ Citron (Citrus medica L.) to Citrus Exocortis Viroid Infection
Source: Viruses. 2019 May 17;11(5):453. doi: 10.3390/v11050453 (PMC6563217; doi:10.3390/v11050453)
Supplement: Supplementary file 1 [file viruses-11-00453-s001.zip › viruses-485757-supplementary/Supplementary files/Table S5.doc]

Table S5. Effect of the genes of plant hormone signal transduction in CEVd-infected citron.

| Gene ID | log_2_FoldChange | *p*-adjusted | Function | Hormone |
| --- | --- | --- | --- | --- |
| Cm093530 | 3.3431 | 2.06E-07 | GH31: Probable indole-3-acetic acid-amido synthetase GH3.1 | IAA |
| Cm240460 | -2.9756 | 0.007295 | GH33: Indole-3-acetic acid-amido synthetase GH3.3 | IAA |
| Cm106810 | 1.7259 | 0.009002 | ARFI: Auxin response factor 9 | IAA |
| Novel00515 | -2.0371 | 0.003995 | hypothetical protein CICLE | IAA |
| Cm001600 | -2.6797 | 3.70E-06 | AX10A: Auxin-induced protein X10A | IAA |
| Cm001800 | -2.8849 | 0.011736 | AX10A: Auxin-induced protein X10A | IAA |
| Cm001780 | -2.6686 | 0.003613 | AX10A: Auxin-induced protein X10A | IAA |
| Cm001820 | -2.0193 | 0.001167 | AX15A: Auxin-induced protein 15A | IAA |
| Cm066850 | -3.8354 | 1.01E-08 | AX22B: Auxin-induced protein 22B | IAA |
| Cm001670 | -2.6998 | 1.36E-05 | SAU19: Auxin-responsive protein SAUR19 | IAA |
| Cm001610 | -2.434 | 4.93E-05 | SAU21: Auxin-responsive protein SAUR21 | IAA |
| Cm001770 | -3.2157 | 5.32E-08 | SAU24: Auxin-responsive protein SAUR24 | IAA |
| Cm011180 | 1.8522 | 0.026777 | SAU32: Auxin-responsive protein SAUR32 | IAA |
| Cm240350 | 2.4681 | 5.12E-05 | GID1B: Gibberellin receptor GID1B | GA |
| Cm198130 | 3.4045 | 1.27E-08 | CCD31: Cyclin-D3-1 | BR |
| Cm011420 | 3.1569 | 9.55E-08 | TIF9: Protein TIFY 9 | JA |
| Cm157630 | 4.425 | 3.17E-08 | TI10A: Protein TIFY 10A | JA |
| Cm220810 | 3.0776 | 3.14E-06 | TI10A: Protein TIFY 10A | JA |

IAA, Auxin; GA, Gibberellin; BR, Brassinosteroid; JA, Jasmonic acid
