# Supplementary material for: Global Transcriptomic Analysis Reveals Insights into the Response of ‘Etrog’ Citron (Citrus medica L.) to Citrus Exocortis Viroid Infection
Source: Viruses. 2019 May 17;11(5):453. doi: 10.3390/v11050453 (PMC6563217; doi:10.3390/v11050453)
Supplement: Supplementary file 1 [file viruses-11-00453-s001.zip › viruses-485757-supplementary/Supplementary files/Table S6.doc]

Table S6. Effect of CEVd infection on the expression of citron genes in RNA silencing pathway.

| Gene ID | log_2_FoldChange | *p*-adjusted | Function |
| --- | --- | --- | --- |
| Cm146130 | 1.7597 | 0.011109 | DCL2: Endoribonuclease Dicer homolog 2 |
| Novel01970 | 3.8296 | 1.55E-05 | RDR1: RNA-dependent RNA polymerase 1 |
| Novel01971 | 3.7942 | 0.00021 | RDR1: RNA-dependent RNA polymerase 1 |
| Cm225400 | 2.0926 | 0.003689 | RDR1: RNA-dependent RNA polymerase 1 |
| Cm196770 | 1.8243 | 0.013878 | AGO2: Protein argonaute 2 |
| Cm010810 | 2.0944 | 0.001366 | AGO7: Protein argonaute 7 |
| Cm260670 | 3.1775 | 2.06E-07 | SDE3: RNA helicase SDE3 |
