# Supplementary material for: Global Transcriptomic Analysis Reveals Insights into the Response of ‘Etrog’ Citron (Citrus medica L.) to Citrus Exocortis Viroid Infection
Source: Viruses. 2019 May 17;11(5):453. doi: 10.3390/v11050453 (PMC6563217; doi:10.3390/v11050453)
Supplement: Supplementary file 1 [file viruses-11-00453-s001.zip › viruses-485757-supplementary/Supplementary files/Table S7.doc]

Table S7. Validation of RNA-seq results by RT-qPCR.

| Pathogen | Gene ID | Gene | log_2_FoldChange | |
| --- | --- | --- | --- | --- |
|  |  |  | RNA-seq | RT-qPCR |
| CEVd | Cm141580 | CAHC | -1.4445 | -1.465 |
|  | Cm246570 | PAE8 | -1.7272 | -1.2825 |
|  | Cm020020 | PLY5 | -3.3112 | -2.4267 |
|  | Cm276170 | LECR | 2.0440 | 0.9175 |
|  | Cm146130 | DCL2 | 1.7597 | 1.3425 |
|  | Cm260670 | SDE3 | 3.1775 | 3.0367 |
|  | Cm125010 | MGL | 2.1604 | 1.6767 |
|  | Cm099990 | PCNA | 1.5573 | 1.4933 |
|  | Cm261020 | GSTX2 | 3.5991 | 2.8967 |
|  | Cm310000 | SAG12 | 6.0056 | 4.4000 |
|  | Cm240620 | PR4B | 5.5025 | 3.5075 |
|  | Cm240350 | GID1B | 2.5613 | 1.2933 |
